# Adaptive Changes of Glioblastoma Cells Following Exposure to Hypoxic (1% Oxygen) Tumour Microenvironment

**DOI:** 10.3390/ijms20092091

**Published:** 2019-04-28

**Authors:** Ahmed Musah-Eroje, Sue Watson

**Affiliations:** 1 Division of Cancer and Stem Cells, Cancer Biology, University of Nottingham, Nottingham NG7 2UH, UK; sue.watson@nottingham.ac.uk; 2 School of Life Sciences, University of Bedfordshire, Luton LU1 3JU, UK

**Keywords:** glioblastoma, hypoxia, CD133, tumour microenvironment, personalised treatment

## Abstract

Glioblastoma multiforme is the most aggressive and malignant primary brain tumour, with a median survival rate of between 15 to 17 months. Heterogeneous regions occur in glioblastoma as a result of oxygen gradients which ranges from 0.1% to 10% in vivo. Emerging evidence suggests that tumour hypoxia leads to increased aggressiveness and chemo/radio resistance. Yet, few in vitro studies have been performed in hypoxia. Using three glioblastoma cell-lines (U87, U251, and SNB19), the adaptation of glioblastoma cells in a 1% (hypoxia) and 20% (normoxia) oxygen microenvironment on proliferation, metabolism, migration, neurosphere formation, CD133 and VEGF expression was investigated. Compared to cells maintained in normoxia (20% oxygen), glioblastoma cells adapted to 1% oxygen tension by reducing proliferation and enhancing metabolism. Both migratory tendency and neurosphere formation ability were greatly limited. In addition, hypoxic-mediated gene upregulation (CD133 and VEGF) was reversed when cells were removed from the hypoxic environment. Collectively, our results reveal that hypoxia plays a pivotal role in changing the behaviour of glioblastoma cells. We have also shown that genetic modulation can be reversed, supporting the concept of reversibility. Thus, understanding the degree of oxygen gradient in glioblastoma will be crucial in personalising treatment for glioblastoma patients.

## 1. Introduction

Glioblastoma multiforme is the most aggressive primary brain tumour classified as a grade IV astrocytoma by World Health Organization (WHO) [1,2]. Despite multimodal treatment with tumour resection surgery based on MRI image analysis, radiotherapy, and treatment with temozolomide, the median survival rate ranges from 15 to 17 months [3,4].

Although the tumour microenvironment consists of a myriad of elements [5], oxygen tension has emerged as an important microenvironmental factor in cancer treatment [6]. Glioblastoma is a heterogeneous tumour [7,8] with a subpopulation of cells at different oxygen levels [9,10,11]. Variation in oxygen concentration can be used to determine tumour heterogeneity [12].

In tumours, the oxygen tension to which cells are exposed is lower than the atmospheric tension used to culture cells in the lab [13]. Determining the appropriate oxygen tension to culture cells in vitro is of utmost importance if reliable translational data can be obtained from in vitro experiments [13].

As glioblastoma grow rapidly, vascularization becomes inadequate, leading to tumour areas with poor oxygen supply [14]. Oxygen tension in glioblastoma in vivo ranges from 0.1% to 10% [14,15]. If the oxygen supply to a tumour microenvironment is reduced, as in the case of hypoxia, there will be poor delivery of therapy to the tumour cells, which will eventually reduce the efficacy of the therapy [16].

Tumour hypoxia increases with increasing WHO grading of gliomas, with grade 4 tumours being associated with severe hypoxia, while grade 2 tumours are associated with modest cellular hypoxia [14]. Hence, hypoxia could be used as a suitable biomarker for the prediction of poor prognosis and highly malignant glioma [17].

Hypoxia has been shown to elevate the expression of cancer stem cell marker, CD133, and induces tumour aggressiveness [18,19,20]. Hypoxia also increases the tempo of evolution in the peri-necrotic niche [21]. The aggressive phenotype of glioblastoma that leads to tumour migration and invasion, as well as loss of apoptosis, is thought to be influenced by hypoxia [22,23]. In addition, radioresistance is also thought to result from hypoxic regions of tumours [24]. 

Hypoxic stress not only regulates tumour immunogenicity, chemoresistance, and plasticity [20,25,26], it also promotes the invasion of healthy brain tissue, thereby constituting a major concern for glioblastoma patients [27]. When tumour cells are in the hypoxic condition, they present several altered phenotypes which include high metastasis tendency and instability of the genome [28].

A subpopulation of tumour cells, glioma stem-like cells (GSCs), have been implicated in tumour recurrence and sustenance [29]. Stem cell fraction in glioblastoma is increased under hypoxic conditions, as is the induction of Notch pathway ligands and targets [30]. Additionally, hypoxia plays a key role in the maintenance of side population signature genes that are overexpressed in hypoxic niches [10].

In the present study, we sought to understand how glioblastoma cells adapt to 1% oxygen tension and whether or not hypoxic-mediated gene expression will be reversed when cells are removed from a hypoxic microenvironment. Our results strongly indicate that glioblastoma cells adapted to 1% oxygen tension by reducing proliferation and enhancing metabolism. Both migratory tendency and neurosphere-forming ability were greatly limited. In addition, hypoxic-mediated gene upregulation (CD133, OCT4 and VEGF) was reversed when cells were re-oxygenated.

Taken together, the current results suggest that hypoxia plays a pivotal role in the adaptation of glioblastoma to the tumour-microenvironment. Understanding the degree of oxygen gradient in glioblastoma will be crucial in personalising treatment for glioblastoma patients.

## 2. Results

### 2.1. Glioblastoma Cells Adapt to Hypoxia (1% Oxygen) Tumour Microenvironment by Reducing Proliferation and Enhancing Metabolism

**Figure 1:**
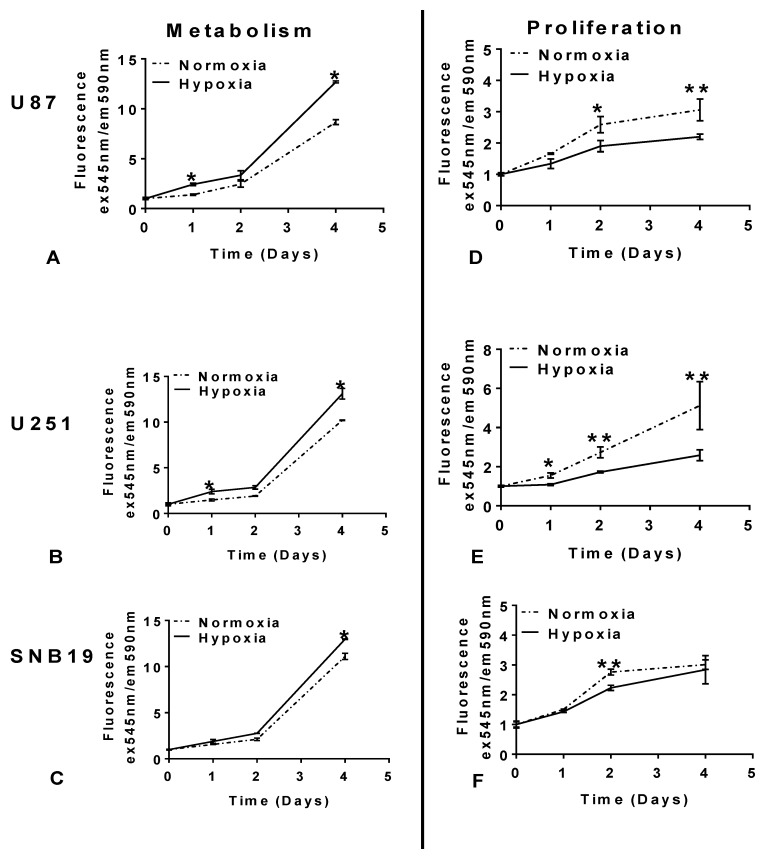
Exposure to hypoxia (1% oxygen) enhances metabolism but not proliferation. Glioblastoma cell lines- U87, U251 andSNB19, were setup with seeding density of 2000 cells per well in a 96-well plate. On the same day of setup, a day 0 reading was taken after cells had settled and a set of cells was transferred to hypoxia (un-dotted), while the control cells were maintained in normoxia (dotted). Metabolism was assessed with AlamarBlue (**A**–**C**), while proliferation was measured with the CyQUANT cell assay (**D**–**F**). The cells were read at the indicated times. The error bar represents standard error from 3 independent experiments for U87, U251, and *n* = 2 for SNB19 cells. Student’s *t*-test from Prism7 was used for statistical comparison. * *p*  <  0.05, ** *p*  <  0.01.

To determine the effect of hypoxia on proliferation and metabolism over time, U87, U251, and SNB19 cell lines were cultured in hypoxic conditions and compared with cells maintained in normoxia for 4 days. We observed that while metabolism was enhanced in hypoxia, it was reduced in normoxia (Figure 1A–C). In contrast, while proliferation was enhanced in cells maintained in normoxia, it was reduced in hypoxia (Figure 1D–F). Our findings suggest that glioblastoma cells adapt to hypoxic (1% oxygen) microenvironment by enhancing metabolism while limiting proliferation.

### 2.2. Glioblastoma Cells Adapt to Hypoxia (1% Oxygen) Tumour Microenvironment by Inhibiting Migration

**Figure 2:**
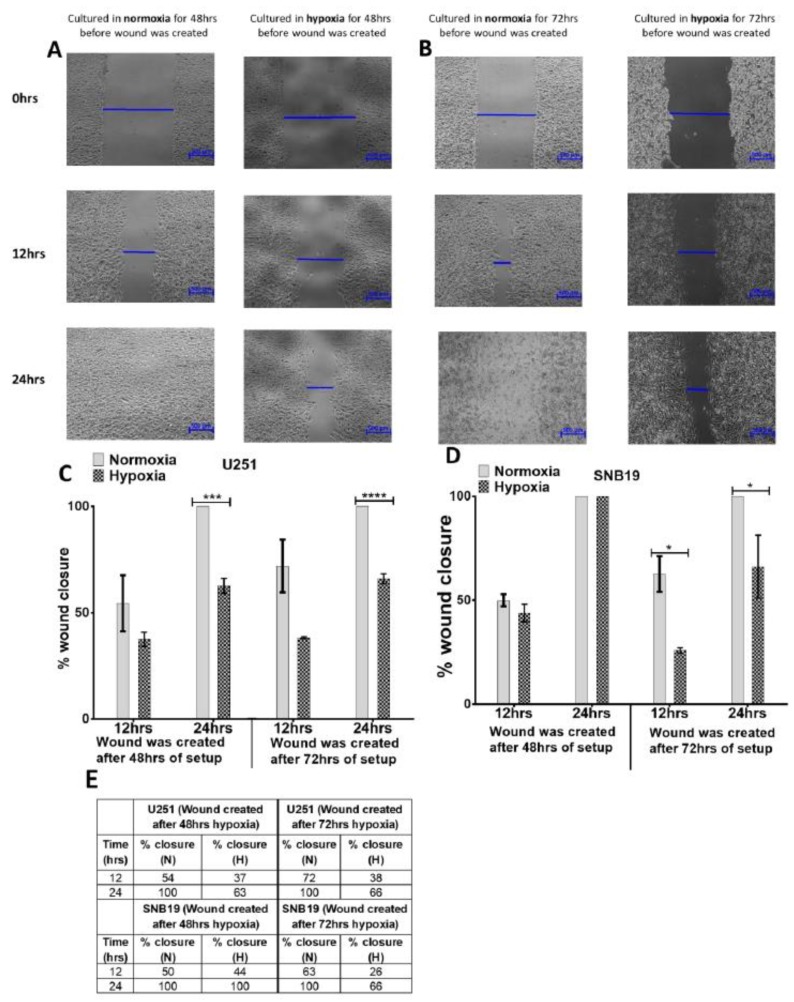
Exposure to hypoxia reduces migration in glioblastoma cells. Representative micrograph of the effect of hypoxia on migration in U251 glioblastoma cell line after 48 h (**A**) and 72 h (**B**) exposure to hypoxia. U251 cells were seeded with a seeding density of 1,000,000 c/w in a 6-well plate. The cells were either maintained in normoxia (20% oxygen)—left side—or hypoxia (1% oxygen)—right side. At either 48 h or 72 h of culture in either normoxia or hypoxia, a wound was created with a P200 tip. Pictures were taken with a Nikon microscope at 0 h, 12 h, and 24 h after the wound was created. The photomicrographs show the migration of U251 cells into the scratched area. (**C**,**D**) Graphical representation of percentage wound closure in the U251 (**C**) and SNB19 (**D**) cell lines. The closure of the wound was calculated as a percentage relative to the wound created at 0 h of wound creation (**E**) Percentage closure of wound after 48 h and 72 h exposure to hypoxia in U251 and SNB19 cell lines. Picture is representative of experiment repeated at least 3 times. * *p*  <  0.05, *** *p*  <  0.001, **** *p*  <  0.0001. Scale bar = 500 µm.

To investigate whether exposure to hypoxia alters the migratory tendency of glioblastoma cells, we performed a scratch-wound assay after exposing U251 and SNB19 cells to hypoxia for 48 h and 72 h respectively. The wound created after 48 h of exposure to hypoxia in U251 and SNB19 was closed 24 h after wound creation in the SNB19 cells but not the U251 cells (Figure 2). Furthermore, when cells were exposed to hypoxia for 72 h before the wound was created, only wounds created in control cells were closed at 24 h (Figure 2). These data suggest that the migratory tendency of glioblastoma cells, as measured by the ability to close the wound, decreased as exposure to hypoxia increased (Figure 2). Conversely, cell migration was markedly reduced after 72 h exposure to 1% oxygen in both cell lines (Figure 2B–E). This was also confirmed in the U87 cells (Figure S2).

### 2.3. Glioblastoma Cells Adapt to Hypoxia (1% Oxygen) Tumour Microenvironment by Limiting Neurosphere Growth

**Figure 3:**
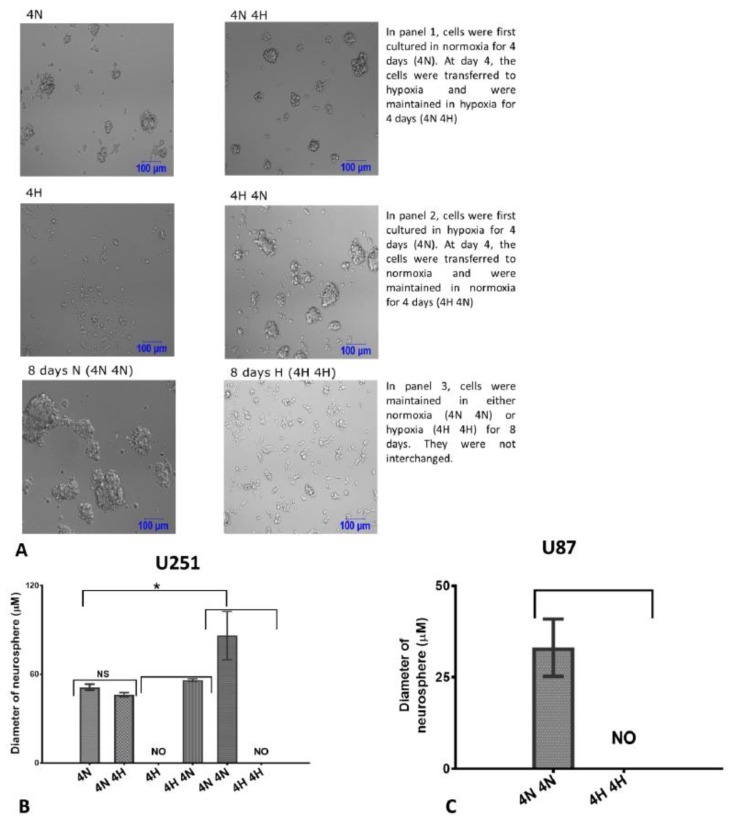
Hypoxia (1% oxygen) impairs the formation of neurospheres: (**A**) 1% oxygen tension impairs the formation of neurospheres. U251 cells were cultured as neurospheres with seeding density of 20,000 cells/well in a 24-well plate. At day 0 of setup, indicated groups were either maintained in normoxia or transferred to hypoxia. The condition of each group is indicated beside the pictures. The pictures are representative of experiment conducted at least 3 times. Scale bar = 100 µm. *n* = Normoxia, H = Hypoxia, D = Day. (**B**,**C**) Diameter of neurospheres formed in U251 (**B**) and U87 (**C**) following exposure to hypoxia: a Nikon confocal microscope was used to measure the width of neurospheres at the indicated days. The error bar indicates the average from two independent experiments. NS = Not significant, NO = Not obtained. * *p* < 0.05.

To understand how the exposure to hypoxia affects the formation of neurospheres, we cultured U251 cells (the U251 cells were used as they readily form neurospheres due to their aggressiveness, while the SNB19 cells do not form neurospheres) under conditions appropriate for the formation of neurospheres in 3 groups): (1) Cells were first maintained in normoxia for 4 days. After 4 days in normoxia, they were transferred to hypoxia and observed for the next 4 days (Figure 3A, panel 1). (2) Cells were first maintained in hypoxia for 4 days. After 4 days in hypoxia, they were transferred to normoxia and observed for the next 4 days (Figure 3A, panel 2). (3) Cells were either maintained for eight days in normoxia or hypoxia respectively (Figure 3A, panel 3). Strikingly, we first observed that hypoxia impaired the formation of neurospheres even though cells were cultured in a stem cell medium (Figure 3A, panels 2 and 3). Interestingly, cells cultured in normoxia for 8 days formed neurospheres while cells maintained in hypoxia did not form neurospheres (Figure 3A, panel 3, Figure 3B). This was also confirmed in the U87 cell lines (Figure 3C). In addition, when cells were first exposed to hypoxia for 4 days, neurospheres were not formed. However, when they were transferred to normoxia for another 4 days, they formed neurospheres (Figure 3A, panel 2). There was no significant difference in the size of neurospheres formed when cells were first maintained in normoxia before they were transferred to hypoxia (Figure 3A, panel 1, Figure 3B), although the spheres were slightly smaller when they were transferred to hypoxia (Figure 3B). This indicates that 1% oxygen tension impairs the formation of neurospheres.

### 2.4. Hypoxic-Mediated Upregulation of CD133 is Reversible

We next ascertained whether glioblastoma cancer stem cell marker, CD133, which is upregulated in hypoxia [20,31], is maintained when cells are removed from the hypoxic environment.

**Figure 4:**
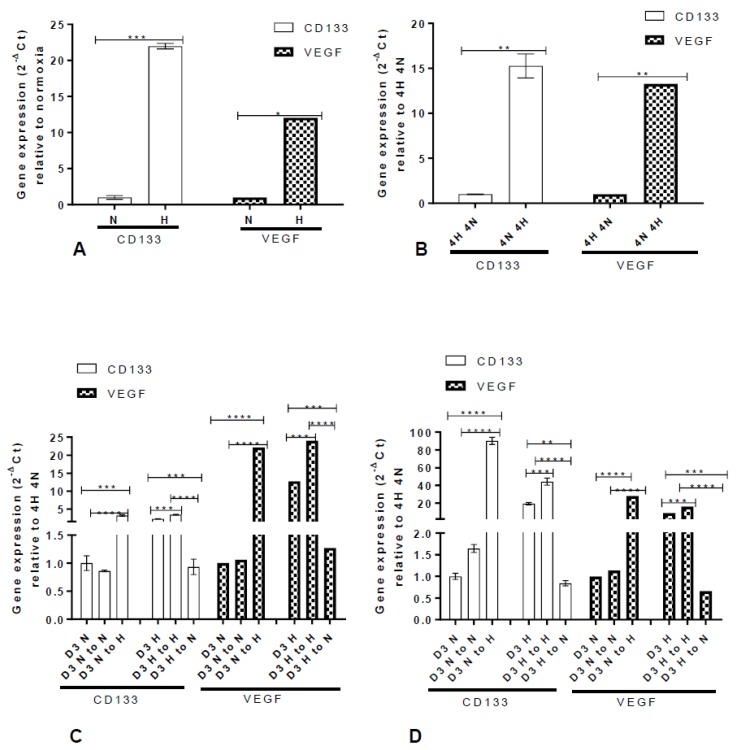
Reversibility of CD133 and VEGF mRNA expression following culture from hypoxia to normoxia. (**A**,**B**) U251 cells were cultured under normoxic (N) or hypoxic (H) conditions. CD133 and VEGF mRNA levels were quantified at day 4 using qRT-PCR (**A**). The cells cultured in hypoxia were subsequently re-oxygenated (20% oxygen) 4H 4N, while cells cultured in 20% oxygen were re-cultured in hypoxia (1% oxygen) 4N 4H. After 4 days, CD133 and VEGF mRNA levels were quantified using qRT-PCR (**B**). U87 (**C**) and U251 (**D**) cells were cultured in normoxia (D3N) and hypoxia (D3H) for 3 days. At day 3 in both conditions, the cells were harvested. Normoxia cells (D3N) were re-cultured in either normoxia (D3N to N) or hypoxia (D3N to H). Likewise, hypoxic cells (D3H) were re-cultured in either hypoxia (D3 H to H) or normoxia (D3 H to H). The cells were maintained for 3 days and mRNA expression of CD133 and VEGF was ascertained with qRT-PCR. The error bars represent an average of 3 independent experiments. One-way ANOVA (Prism7) was used for statistical comparison. ** *P*  <  0.01, *** *P*  <  0.001. **** *P* ˂ 0.0001.

**Figure 5:**
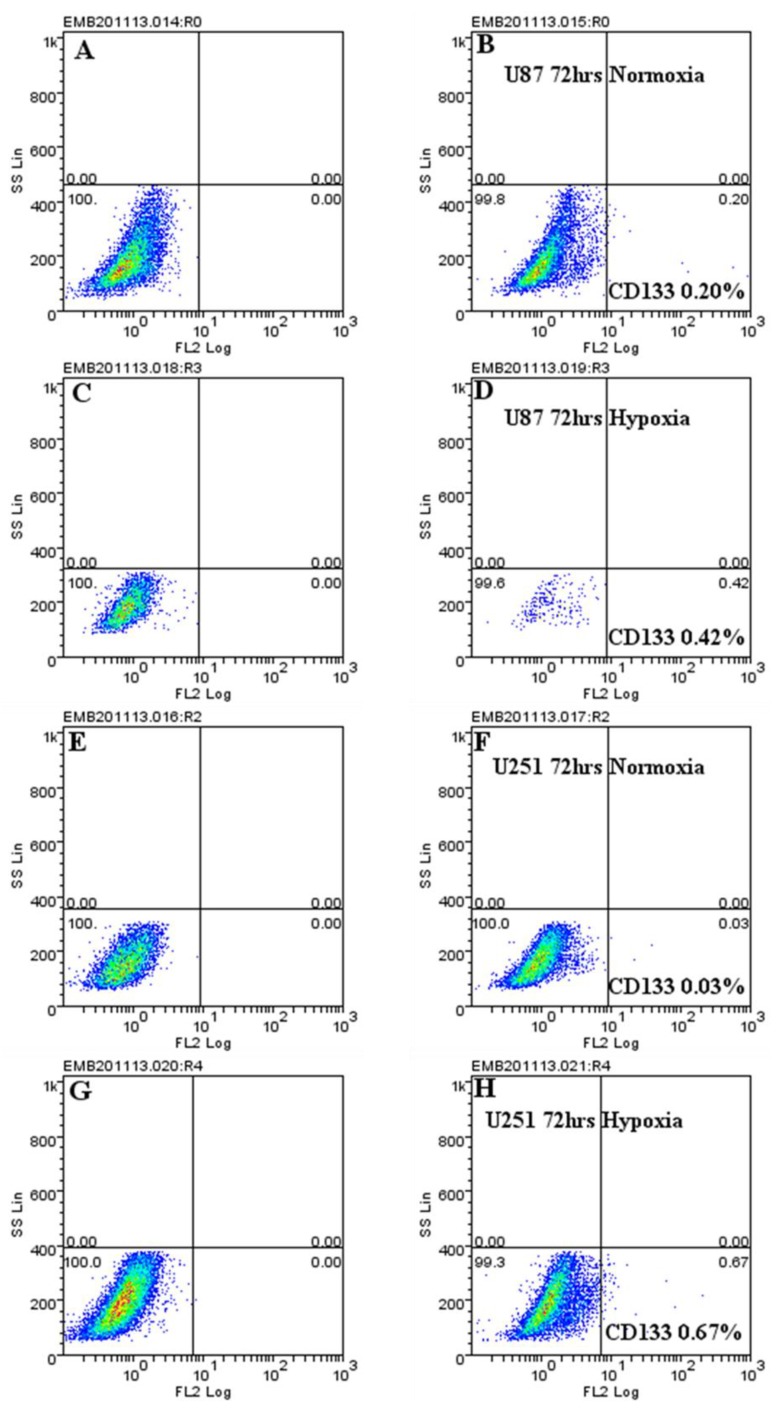
CD133 protein is upregulated under hypoxic conditions. U87 (**A**–**C**), and U251 (**E**–**H**) cells were cultured under normoxic (**A**,**B**,**E**,**F**) and hypoxic (**C**,**D**,**G**,**H**) conditions for 72 h. For both conditions, the total isotype control cell populations (**A**,**C**,**E**,**G**) are presented based on side and scatter properties, and appropriate regions are gated and used to compare cells stained with the anti-CD133 antibody (**B**,**D**,**F**,**H**). The percentages of cells expressing CD133 after 72 h are indicated. The analyses were performed using Weasel software. The results are representative of at least 3 independent experiments.

**Figure 6:**
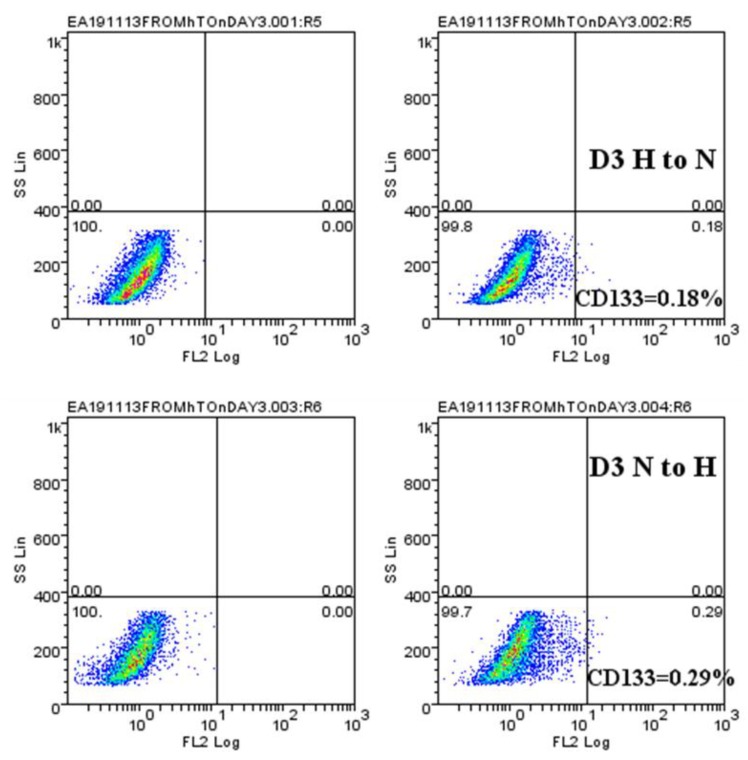
CD133 expression is lost when hypoxic cells are recultured under normoxic conditions. Following exposure of U251 cells to either normoxia (D3 N) or hypoxia (D3 H), the cells were recultured in either normoxia (D3 H to N) or hypoxia (D3 N to H). For both conditions, the total isotype control cell populations are presented based on side and scatter properties, and appropriate regions are gated and used to compare cells stained with the anti-CD133 antibody. The percentages of cells expressing CD133 after 72 h of recultured are indicated. The analyses were performed using Weasel software. The results are representative of at least 3 independent experiments.

When cells were exposed to hypoxia, CD133 mRNA was upregulated (Figure 4A). Similarly, VEGF mRNA, which was used as a positive marker for hypoxia, was upregulated (Figure 4A). However, we observed that both CD133 and VEGF mRNAs returned to baseline when the cells were returned to normoxia (Figure 4B). This was also observed with OCT4 mRNA (Figure S1). To further validate this finding, U87 and U251 cells were cultured in normoxia (D3N) and hypoxia (D3H) for 3 days. At day 3 in both conditions, the cells were harvested, and CD133 gene and protein expression determined. Cells cultured in normoxia (D3N) were re-cultured in either normoxia (D3 N to N) or hypoxia (D3 N to H). Similarly, cells cultured in hypoxia (D3H) were re-cultured in either hypoxia (D3 H to H) or normoxia (D3 H to N) (Figure 4C,D). The cells were then maintained for 3 days and CD133 mRNA and protein and VEGF mRNA expression ascertained (Figure 4 and Figure 5). The results revealed that the CD133 stem cell marker returned to baseline both at the gene and protein level when the cells were moved from a hypoxic environment to normoxia (i.e., re-oxygenation) (Figure 6), confirming the concept of reversibility.

## 3. Discussion

Glioblastoma is an aggressive tumour with the ability to proliferate rapidly and infiltrate the surrounding brain tissue. One of the key features of glioblastoma aggressiveness is the ability to proliferate rapidly and infiltrate the surrounding brain tissue [32].

The results presented here show that oxygen tension affected the metabolism of glioblastoma cells. Under hypoxia, the rate of conversion of resazurin, which is the active ingredient of Alamar blue, to resorufin was greatly enhanced when compared to cells cultured in normoxia. The reducing environment of cells cultured in hypoxia could be useful in converting resazurin to resorufin. Interestingly, in the electron transport chain, resazurin acts as an intermediate electron acceptor [33]. As the electron is accepted, it changes from the blue non-fluorescent oxidized form to the pink fluorescence reduced form [33]. Hence, the conversion of resazurin to resorufin can be used to measure nicotinamide adenine dinucleotide (NADH) [34].

Interestingly, NADH levels are elevated under hypoxia [35,36]. This finding, therefore, indicated that 1% oxygen tension did not impair the activity of mitochondria, and it is in agreement with previous studies [37,38]. Hence, under a hypoxic environment, the metabolism of glioblastoma increases. In contrast, the proliferation of cells cultured in hypoxia was greatly impaired compared to cells maintained in normoxia. Our finding is in agreement with previous data on mouse embryonic stem cell [39], but dissimilar with data from Richards et al. (2016), who showed that pathophysiological hypoxia has minimal effects on the proliferation and survival of glioblastoma [40]. However, in accordance with our finding that cells grow slower in a hypoxic microenvironment, Holzwarth et al. (2010) recently reported with an MTS proliferation assay that hypoxia reduced the proliferation of human multipotent mesenchymal stromal cells, while 20% oxygen tension led to vigorous proliferation [41]. They also found that more cells were at the G_2_/M phase at an oxygen concentration of 20% compared with cells cultured at 1% [41]. In agreement with these findings, an MTT assay revealed that hypoxia slowed down the growth of glioblastoma cells and increased the percentage of cells in the quiescent stage [42]. Indeed, in this study, the Cyquant assay made it possible to precisely assess effects on cell number by measuring the DNA content as against metabolically-based assays which may be influenced by factors unrelated to cell number [43].

There are still conflicting results as to whether hypoxia promotes migration in glioblastoma. Several migration models have been proposed. However, the scratch-wound assay was utilized to assess the migration of glioblastoma cells to understand the role of hypoxia (1% oxygen) in the migration of glioblastoma. In marked contrast to 20% oxygen, 1% oxygen greatly inhibited migration. This is in line with the observation that 1% oxygen tension slowed the migration of fibroblast cells [44]. Vogler et al. (2013) first created a scratch in a monolayer of cells and observed a marked reduction in the overall speed of wound closure in hypoxic cells which was further confirmed in a single cell motility assay [44]. Our finding is not in agreement with Li et al. (2013) and Zigzag et al. (2006), who recently showed that the migration of U87 cells increased in hypoxic conditions [42,45]. The reason for this difference may be attributed to the fact that they used a transwell migration assay which utilizes a chemoattractant rather than the scratch assay used in this study.

The migration of human lung adenocarcinoma cells was facilitated after 20 h of exposure to hypoxia [46]. We evaluated the migration of cells after they were exposed to hypoxia for 48 h and 72 h. With longer exposure to hypoxia, migration was markedly reduced in 1% oxygen when compared with 20% oxygen. Some data have shown that hypoxia facilitates cell migration [46,47,48]. It is possible that the rate of migration of cells during acute exposure to hypoxia is high. However, with prolonged/chronic exposure to hypoxia, migration of cells could be greatly hindered as seen in our study. Recent data has emerged linking hypoxia and the extracellular matrix to tumour metastasis [49], but the current study was performed without the incorporation of extracellular matrix. Nonetheless, since our findings showed that both proliferation and migration decreased in hypoxia, it may not be appropriate to explicitly conclude that the filling of the gap in the scratch assay was as a result of migration. A combination of lower proliferation and lower migration may have been observed. However, to be able to distinguish between migration and proliferation, the latter could be controlled for using mitomycin C [50,51].

It is well established that the hypoxic microenvironment selects for stem-like cancer cells [20,52]. The hypoxic environment is also known to preserve embryonic stem cell phenotype [53]. The need to expand neural stem cells in vitro has led to the development of the neurosphere assay, which is utilized to expand cells that are thought to possess stem-like characteristics [54].

Because the neurosphere assay and hypoxia select for stem like cells, it was reasonable to attempt to culture glioblastoma cells as neurospheres in a hypoxic environment. In fact, a previous study indicated that the growth of U87 cells as neurospheres under 2% oxygen tension promoted their proliferation [55]. The spheres could be expanded and maintained as spheroids through many passages and expressed cancer stem cell marker, CD133 and nestin [55]. However, regions of hypoxia in glioblastoma in vivo vary from 0.1% to 10% [14]. In contrast to 2% oxygen tension, the result from this study revealed that neurospheres were barely formed at 1% oxygen tension. This is in agreement with previous data that showed that 1% oxygen tension was most deleterious to neurosphere survival [37]. Moreover, under 2% oxygen tension, Rosenbert et al. (2018) showed that the size of normoxic spheroids increased over 7 days, while the size of hypoxic spheroids was constant [56], which is consistent with our finding that reduced oxygen tension limits the expansion of neurospheres. The highest proliferation rate for neurosphere formation was obtained at 2.5 and 5% oxygen tension [37]. In contrast with these findings, Chung et al. (2014) revealed that reducing oxygen levels to 1% and 5% had no significant effect on either neurosphere number or size [57]. In their studies, neurospheres were derived from adipose tissue-derived mesenchymal stromal cells.

Even when cells were first allowed to form neurospheres at 20% oxygen, the growth of the spheres was impaired when the cells were transferred to 1% oxygen environment. In line with this, the proliferation of induced pluripotent stem cells (iPS) from human somatic cells was optimal at mild hypoxia in comparison to 1% oxygen [58]. Thus, the finding from the current study suggests that glioblastoma stem cells residing in 1% oxygen tension do not proliferate.

Hypoxia has been widely reported to be a factor in the tumour microenvironment in glioblastoma [10]. Most of the cells generated from the hypoxic niche have been shown to be CD133 positive and resistant to therapy [20,59]. Our result shows a strong hypoxia-induced upregulation of VEGF and CD133 which is a stem cell marker in glioblastoma. Interestingly, the expression of these genes was reversible [60] revealing the plasticity of glioblastoma cells.

Although plasticity in tumours has been well published, the concept of reversibility has been given little attention. If stem cells explicitly express certain genes, these genes should be constantly expressed in a different environment. Our data could imply that the term “stem cells” as used in glioblastoma may be a transient state that is influenced by the microenvironmental conditions—in this case, hypoxia. For instance, as a result of reversibility of CD133, it has been suggested that its usage as a stem cell marker should be revised [60]. Our findings reveal that the last microenvironment determines the molecular signature and not the previous. This will mean that in most situations, tumour cells will express the same genetic signature, as long as they are in a constant microenvironment. When they leave that environment, they will express a genetic signature that will allow them to adapt to their new environment and reverse these signatures if their microenvironment changes. We do not know exactly how long these cells might need to be in an environment to make their genetic signature irreversible. It would be of great interest to know the genetic cues that facilitate their ability to change from one microenvironment to the other. This might be a useful therapeutic target in the future.

Remarkably, our findings have linked oxygen levels to phenotypic and genetic changes in glioblastoma. Understanding the degree of oxygen gradient in glioblastoma will be crucial in personalising treatment for glioblastoma patients. Therefore, we suggest that oxygen gradient should be given more attention in the research on glioblastoma.

## 4. Material and Methods

### 4.1. Cell-Lines

U87 cells, from European Type Culture Collection (ECCC, Salisbury, UK) while U251 and SNB19 cells from National Cancer Institute (Maryland, USA), NCI60, were grown in normoxic (20% oxygen) or hypoxic (1% oxygen) conditions as standard 2D culture and as neurospheres. All standard 2D cells were cultured in RPMI-1640 medium (Invitrogen, Loughborough, UK containing 10% foetal bovine serum and 1% L-glutamine (Sigma-Aldrich, Irvine, UK). The cultures were maintained in a SANYO incubator set at 5% CO_2_ at 37 °C and were expanded when confluent.

### 4.2. Neurosphere Culture

Neurosphere was performed as previously published [20]. Neurosphere cultures were maintained in 128 mL High Glucose Dulbecco’s Modified Eagle’s Medium (Invitrogen, Loughborough, UK) in which 116 mL F12 Ham (Invitrogen, UK) was added and supplemented by 10 mL B27 supplement (Invitrogen, UK), 100 µg/mL FGF (Invitrogen, UK) and 100 µg/mL EGF (Invitrogen, UK), as well as 100 mg/mL Heparin (Sigma-Aldrich, Irvine, UK St. Louis, MO, USA), at 37 °C in a 5% CO_2_ and humidified atmosphere. Cells were seeded in a 24-well plate at a density of 20,000 cells/well.

### 4.3. Alamar Blue and CyQUANT Assays

Alamar Blue was diluted 1:10 and 50 μL of the solution was added to each well. The cells were then allowed to incubate for 1 h. Thereafter, the fluorescent signal was read using a FlexStation II (Molecular Devices, CA, USA) set at to produce excitation at 530–560 nm and detect emission at 590 nm. The CyQUANT assay cell proliferation kit was purchased from Life Technologies and was used to measure cell number as it binds to DNA. The medium from the wells was aspirated and the plates were transferred to a −80 °C freezer. After cells were retrieved from the freezer, they were thawed and the cyQUANT dye was added to lysis buffer. The fluorescence was measured using a Flex station with excitation at 485 nm and emission at 530 nm.

### 4.4. Flow Cytometry

Flow cytometry was performed as previously published [20]. Briefly, after harvesting, glioblastoma cells were spun down in Eppendorf tubes and re-suspended in 80 μL buffer and 20 μL FcR blocker (Miltenyi Biotec, Bisley, UK), after which 10 μL anti-CD133/1 (AC133)-PE antibody (Miltenyi Biotec, Bisley, UK) or a mouse monoclonal IgG-PE isotype control was added according to the manufacturer’s instructions. Next, the cells were analysed using a Beckman Coulter flow cytometer. The resulting data were analysed using the Weasel software (http://www.frankbattye.com.au/Weasel/).

### 4.5. The Culture of Cells in a Hypoxia Chamber

The Invivo2 400 hypoxia workstation (Ruskinn technology LTD, Wales, UK) was used to set oxygen concentration at 1%. The chamber was accessed through an Ezee sleeve and purged with vacuum and gas pedal. The chamber which is set at 5% CO_2_ at 37 °C is attached to a nitrogen cylinder which helps to maintain the oxygen concentration in the chamber.

### 4.6. Scratch Assay (Wound Healing Assay)

Cells were seeded at day 0 such that they became confluent on day 1. When a 6-well plate was used, 1,000,000 cells/well were seeded at day 0. For the 96-well plate, the cells were seeded with 30,000 cells/well at day 0. The monolayer of cells was then scratched in a straight line with a p200 pipet tip. The debris of cells was removed by washing the cells with a gentle stream of medium, and fresh medium was added to respective wells. Pictures were then taken with a Nikon microscope at day 0 (baseline reading) and subsequently to the end of the study. To acquire pictures at the same point, the microscope was used to set specific coordinates that were re-used for each acquisition. To analyse the pictures, the software from the microscope was used to make a line from one side of the scratch to the other. Multiple lines were drawn on the monolayers of cells and an average of the width was calculated.

### 4.7. Quantitative Real-Time PCR

Gene expression was assessed using real-time RT-PCR and data expressed relative to the housekeeping gene, HPRT, as previously reported [20]. The expression of CD133, VEGF and OCT4, which are commonly used to define the CSC-like population in brain tumours, were detected using SYBR Green (Eurogentec) and calculated using the 2^−∆∆Ct^ method. The primer sequences used were: CD133 forward: 5′-CAATCTCCCTGTTGGTGATTTG-3′; and CD133 reverse: 5′-ATCACCAGGTAAGAACCCGGA-3′; VEGF forward: 5′-CCAAGTGGTCCCAGGCTGCA-3′; reverse: 5′-TGGATGGCAGTAGCTGCGCT-3′. OCT4 forward: 5′-GTTGGAGAAGGTGGAACCAA-3′; and OCT4 reverse: 5′-CTCCTTCTGCAGGGCTTTC-3′.

### 4.8. Statistics

Student’s *t*-test from GraphPad Prism, version 7, was used to analyse all data. Data were analysed with either *t*-test or one-way ANOVA (Tukey’s multiple comparison test).

## Supplementary Materials

The following are available online at https://www.mdpi.com/1422-0067/20/9/2091/s1.
