# Supplementary material for: Adaptive Changes of Glioblastoma Cells Following Exposure to Hypoxic (1% Oxygen) Tumour Microenvironment
Source: Int J Mol Sci. 2019 Apr 28;20(9):2091. doi: 10.3390/ijms20092091 (PMC6539006; doi:10.3390/ijms20092091)
Supplement: Supplementary file 1 [file ijms-20-02091-s001.pdf]

Supplementary

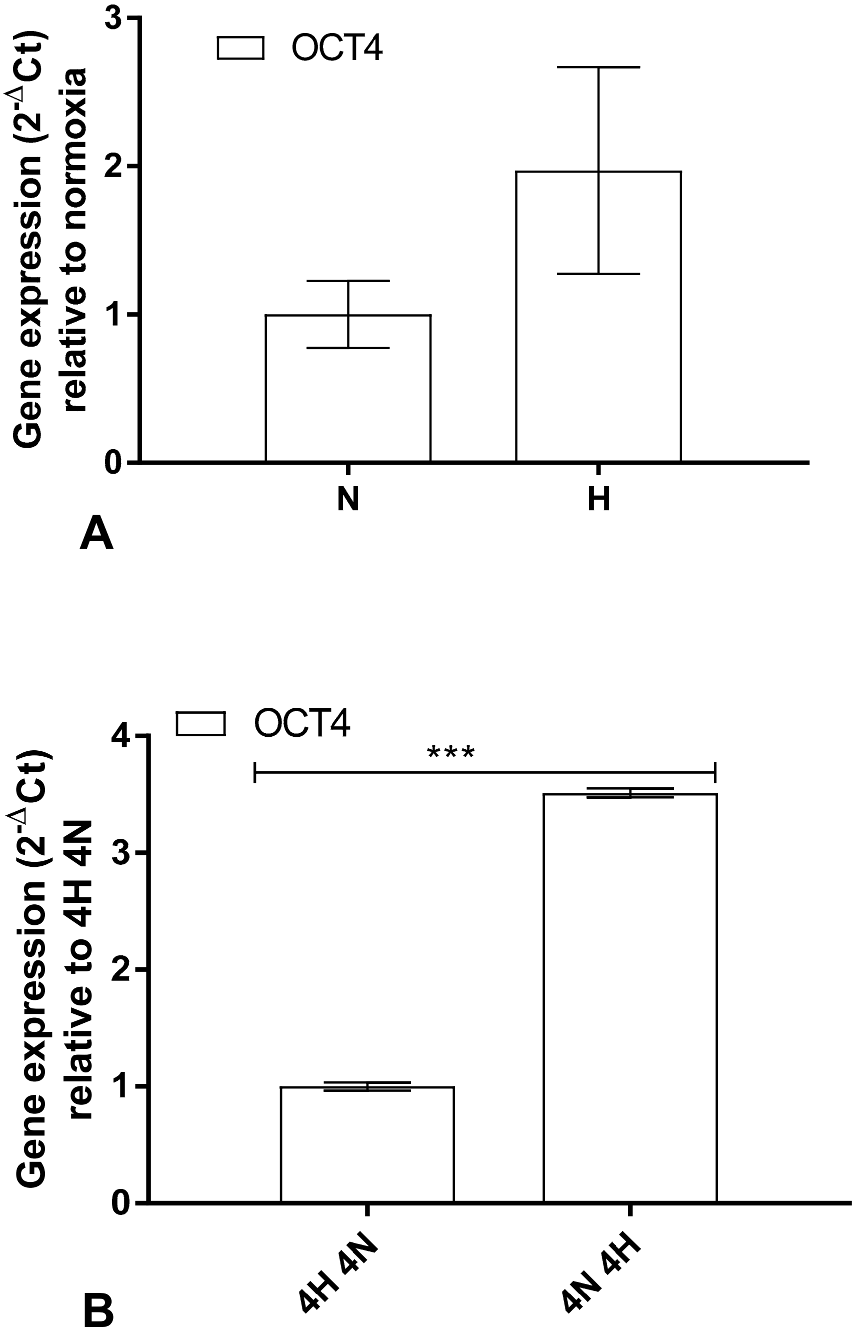

Figure S1. 4 Reversibility of OCT4 mRNA expression following culture from hypoxia to normoxia. U251 cells were cultured under normoxic (N) or hypoxic (H) conditions. OCT4 mRNA levels were quantified at day 4 using qRT-PCR **(A)**. The cells cultured in hypoxia were subsequently re-oxygenated (20% oxygen)-4H 4N, while cells cultured in 20% oxygen were re-cultured in hypoxia (1% oxygen) - 4N 4H). After 4 days, OCT4 mRNA levels were quantified using qRT-PCR **(B)**.

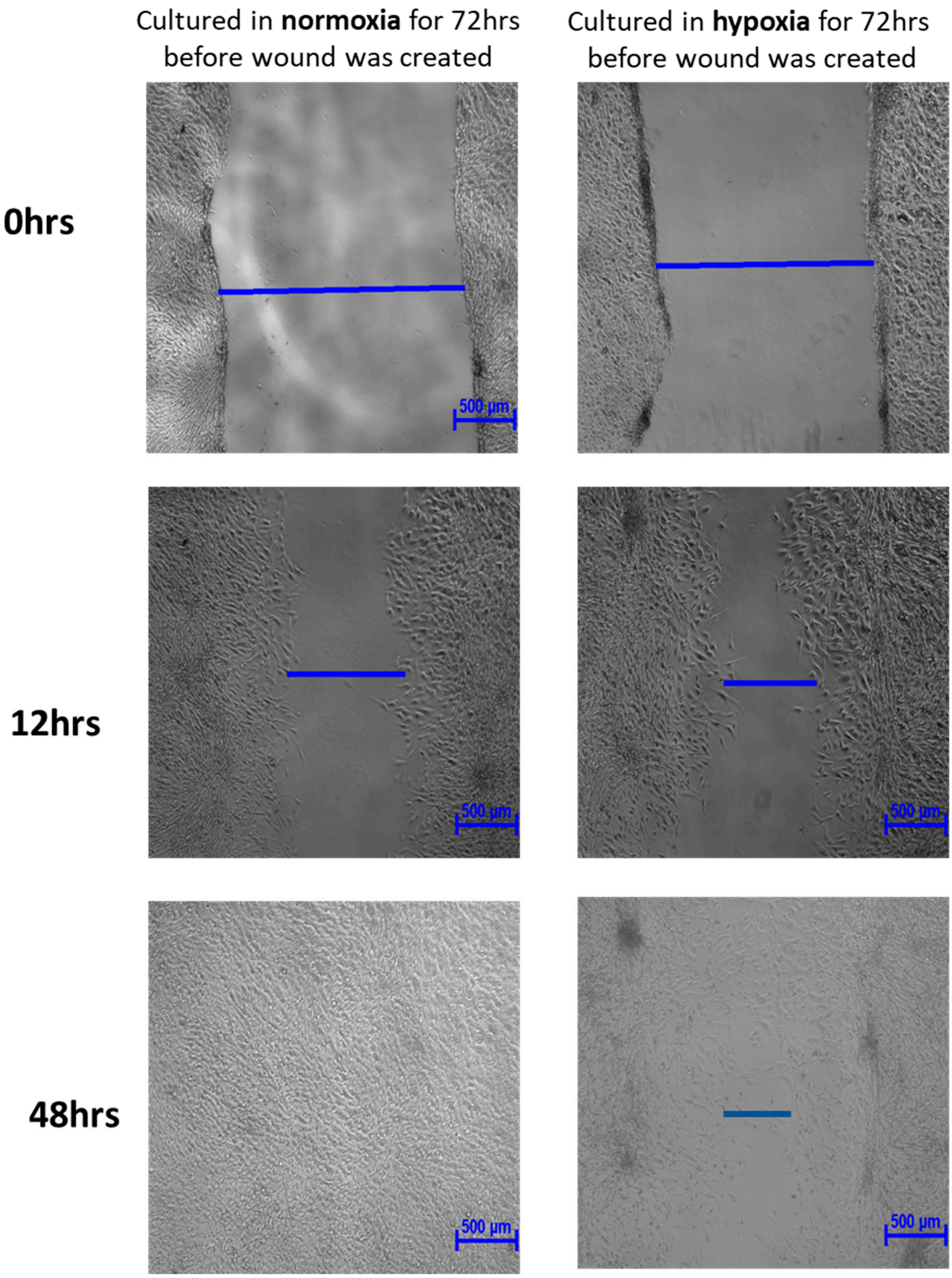

Figure S2. Exposure to hypoxia reduces migration in glioblastoma cells. Representative micrograph of the Effect of hypoxia on migration in U87 glioblastoma cell line after 72hrs exposure to hypoxia. U87 cells were seeded with a seeding density of 1,000,000 c/w in a 6-well plate. The cells were either maintained in Normoxia (20% oxygen)-left side or hypoxia (1% oxygen) - right side. At 72hrs of culture in either normoxia or hypoxia, a wound was created with a P200 tip. Pictures were taken with a Nikon microscope at 0, 12hrs, and 48hrs after the wound was created. The photomicrographs show the migration of U87 cells into the scratched area. Picture is representative of experiment repeated at least 2 times. Scale bar = 500µm.
